# Supplementary material for: Prevalence of falls and comparison of health-related physical fitness factors between different faller categories among institutionalized older adults in Kandy District of Sri Lanka
Source: PLoS One. 2024 Feb 20;19(2):e0297946. doi: 10.1371/journal.pone.0297946 (PMC10878510; doi:10.1371/journal.pone.0297946)
Supplement: Attachment — Submitted filename: Response to Reviewers.pdf [file pone.0297946.s002.pdf]

Antimo Moretti,
Academic Editor,
PLOS ONE

Dear Sir,

I'm grateful for the comments you have given for my paper submitted on the topic "Prevalence of falls and comparison of health-related physical fitness factors between different faller categories among institutionalized older adults in Kandy District of Sri Lanka". I carefully went through the comments made by you as well as by the reviewers. Herewith I'm submitting the point to point response for all the comments raised.

Reviewer comments	Response
**Academic editor**	
1. Please ensure that your manuscript meets PLOS ONE's style requirements, including those for file naming.	PLOS ONE's style requirements were followed.
2. We note that you have indicated that data from this study are available upon request. PLOS only allows data to be available upon request if there are legal or ethical restrictions on sharing data publicly.	The data sheet of the study will be submitted along with the revised manuscript.
3. PLOS requires an ORCID iD for the corresponding author in Editorial Manager on papers submitted after December 6th, 2016.	The ORCID ID of the corresponding author is updated in the Editorial Manager Account.
**Reviewer 01**	
1) The introduction talks about quality of life, depression and other aspects of the institutionalized elderly. The concept is far from the focus of the title and is not related to the risk of falls. I recommend learning more about the concepts.	- The statement regarding sleep disorders is removed from the introduction.<br>- Information about quality of life and risk of falls in included.
2) Describe the 12 homes for the elderly and the enlistment in the materials and methods but also in the introduction. I advise you to mention it only in the materials and methods.	Addressed
3) You describe the names of 8 homes for the elderly, but in the results I only find 7.	The stated number of Elders' homes in Material and Methods is seven and it is in line with (same as) the number of elderly homes stated in results.
4) This statement in the results should be more precise and describe only the scales "The results in Table 2 revealed that there was a significant difference in BMI, cardiovascular	Addressed

lower body strength and flexibility across the three fall categories. Further,<br>The Scheffe test was conducted for pairwise comparison of the factors shown statistically significant difference". Better to make the comments in discussion.	
5) Table 3 is repetitive. The categories are repeated several times.	Addressed
6) in the discussion the statement that institutionalization correlates to falls is incorrect as there is no group of non-institutionalized elderly comparisons	That statement is removed from the discussion.
**Reviewer 02**	
Title: well done.	Thankful for the comment.
Abstract: see methods' comment, I would do the same changes about measures description.	Abstract revised to incorporate the comment and to maintain the word limit.
Introduction: I would had that there have been different study investigating the correlation between different diet/ integration and the risk of falls (for example I would suggest to cite this study DOI: 10.1007/s40520-021-01977-x).	The objective of the study did not include, exploring diet patterns and/or its association with risk of falls among older adults, therefore, information about diet patterns is not included
Methods<br>Measures: in this section I would be more specific describing which of the variable (bioelectrical impedance analysis, 2 minute walk test, 30 second sit to stand test, hand grip strength measure, chair sit and reach test, and back scratch test) is the measure of each outcome (Body composition, cardiovascular endurance, lower body strength and endurance, upper body strength, lower body flexibility, upper body flexibility).	Addressed
Results, discussion, and conclusion: well described.	Thankful for the comment.

Thank you
Yours sincerely,

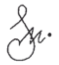

(W.I.S.M.IHALAGE)
